# Skin/mucosa avoidance radiotherapy (SMART) versus conventional volumetric arc‐based radiotherapy (VMAT) for the treatment of head and neck cancer: Dosimetric feasibility study

**DOI:** 10.1002/acm2.14000

**Published:** 2023-05-04

**Authors:** Cheryl Anderson, R Lee MacDonald, Derek Wilke

**Affiliations:** ^1^ Department of Radiation Oncology Nova Scotia Health Authority Halifax Canada; ^2^ Department of Physics and Atmospheric Science Dalhousie University Halifax Canada; ^3^ Department of Radiation Oncology Dalhousie University Halifax Canada

**Keywords:** mucosa, oral cavity, skin sparing

## Abstract

**Background:**

Intensity modulated radiotherapy (IMRT) for head and neck cancer has led to a reduction in radiotherapy doses to normal tissues, like the salivary glands, while maintaining high rates of local control. Oral mucosal and skin toxicity is still a major source of treatment—related morbidity, occurring in most patients.

**Purpose:**

We conducted a dosimetric feasibility study with the goal of creating a methodology that could theoretically reduce the dose of radiation to skin and oral mucosa, while maintaining comparable avoidance of other organs at risk, and planning target volume (PTV) coverage.

**Methods:**

The clinical plans of patients treated previously were replanned using coplanar VMAT arcs on a TrueBeam STx using the photon optimizer (PO) version 15.6 and the Acuros XB dose calculation algorithm. Comparisons were made between three methodologies: “Conventional,” “Skin Sparing” and a skin/mucosa avoiding (“SMART”) technique, with dose metrics being compared using analysis of variance, with a Bonferroni correction to account for multiple pairwise comparisons. The maximum grade of mucositis and radiation dermatitis during treatment was correlated to different dose‐volume metrics to predict what could be clinically meaningful.

**Results:**

Sixteen patients met the study criteria and were replanned using the skin sparing and SMART techniques. Maximum doses to the skin sparing structure were reduced from 64.2 Gy to 56.6 and 55.9 Gy, in the skin sparing and SMART plans (*p* < 0.0001), and mean doses reduced from 26.7 Gy to 20.0 and 20.2 Gy, respectively (*p* < 0.0001). Maximum doses to the oral cavity structure were not reduced by either technique, however mean dose to the oral cavity structure was reduced from 39.03 Gy to 33.5 Gy by the SMART technique (*p* < 0.0001). There was a slight reduction in PTV_High coverage by the V95% in the SMART plans (99.52% vs. 98.79%, *p* = 0.0073), and a similar slight reduction in PTV_Low coverage by the V95% by both the skin sparing and SMART plans (99.74% vs. 97.89% vs. 97.42%, *p* < 0.0001). Maximum doses to organs at risk were not statistically different between techniques. Dose to oral cavity and maximum grade experienced during radiotherapy correlated. The Spearman correlation coefficient for dose to 20%, 50%, and 80% of the volume of oral cavity was 0.5 (*p* = 0.048), 0.64 (*p* = 0.007), and 0.62 (*p* = 0.010), respectively. Skin toxicity grade was only found to be correlated with the D20% of the skin sparing structure (Spearman correlation coefficient of 0.58, *p* = 0.0177).

**Conclusion:**

The SMART technique appears to be able to reduce maximum and mean skin dose, as well as mean oral cavity doses, while only slightly reducing PTV coverage, with acceptable OAR doses. We feel the improvements warrant investigation in a clinical trial.

## 1 INTRODUCTION

With over 5400 new cases and 1500 deaths predicted in Canada in 2020, Head and Neck cancer continues to be a challenging cancer site.^1^ Despite the introduction of intensity modulated radiation therapy (IMRT) that can dramatically spare normal structures of the head and neck, like the salivary glands, head and neck radiotherapy continues to possess considerable side effects, leading to a dramatic decline in quality of life.^2^ The incidence of grade >2 skin toxicity is reported to be between 86% and 92%, and the incidence of all grades of mucositis to be 80%, with approximately 33% of patients experiencing grade > 3 mucositis, clearly indicating a need to improve upon current treatment.^3^ These toxicities are not insignificant. They can lead to radiotherapy treatment delays, early cessation of concurrent chemotherapy, infection, hospitalization, the need for a feeding tube, and treatment‐related death in a very small proportion of patients.^3^

In addition to the notable advancements in H&N radiotherapy in the last two decades, we believe that further improvements can be made by refining the sparing of normal tissues, namely skin and mucosal surfaces in radiation treatment plans. Whereas there is very careful attention to delivering safe doses of radiation to spinal cord, brainstem, optic structures, salivary tissue, mandibular bone, and brain, currently there is only cursory attention made to reducing radiation dose to the oral cavity and skin that is not adjacent to high‐dose targets.

Previous planning studies by Lee and Arnould have shown that reduction in skin dose is achievable, although does not appear to have been widely adopted. The study by Lee demonstrated that the volume of skin receiving 45 Gy could be reduced by 20%.^4^, ^5^ NRG‐HN002, a randomized, phase 2 study of radiation dose de‐intensification, currently recommends a mean oral cavity dose of 32 Gy.^6^ However, the approach to oral cavity sparing has evolved considerably over time, as the dominant radiation technique has evolved from three‐dimensional radiotherapy, to step‐and‐shoot intensity modulated radiotherapy (SS‐IMRT) to volumetric arc‐based techniques (VMAT) over the last two decades. A publication by Lee^7^ in 2006 indicated that IMRT compared to a concomitant boost technique did reduce grade 3 mucositis from 72% to 66%, but the patients who received IMRT earlier in that cohort did not have the oral cavity contoured as an organ at risk, but subsequently did, at which point, they tried to achieve a mean oral cavity dose ≤35 Gy. Another study by Sanguineti^8^ correlated the dose to the oral mucosa (as delineated from the hard palate to the bottom of the hypopharynx) and tried to limit the dose to the oral mucosa that was outside the PTV to 30 Gy or less, and found that the dose to 21 cc of the oral cavity was predictive of grade 3 or greater mucositis. A more contemporary study by Cui in 2019,^9^ compared the acute effects of SS‐IMRT to VMAT, and were able to reduce the mean oral cavity dose from 36.57 to 32.77 Gy (*p* < 0.01), however this did not translate into a reduction in grade of mucositis, but the VMAT cohort did have a significantly lower rate of dysphagia and xerostomia, as the VMAT technique also provided lower mean doses to parotid glands, larynx, pharyngeal constrictors, and esophagus.

The work outlined here aims to further reduce radiation dose to the oral cavity and skin, while maintaining full dose to tumor. We refer to this herein as skin/mucosa avoidance radiotherapy (SMART).

## 2 METHODS

After Institutional research ethics board approval of the project, 16 consecutive patients were identified that had been treated within one calendar year with radiation for H&N cancer. All included patients completed their course of radiotherapy receiving a dose of 70 Gy in 35 fractions. Of the 16 test cases, the primary site was Larynx in two cases, Nasopharynx in three cases, and the remainder had cancers arising from the oropharynx. The weekly skin and oral mucositis toxicity scores (Common toxicity Criteria adverse events version 5.0 (CTCAE v5.0) {7} were extracted from the electronic record and verify system (ARIA, Varian Medical systems, Inc., Palo Alto, USA) to investigate which organs at risk were predictive for mucositis, and correlations estimated by Spearman's correlation coefficient.

The 16 patients selected for use in this study were inversely planned in Eclipse (Varian Medical Systems, Palo Alto, USA) treatment planning system using coplanar VMAT arcs on a TrueBeam STx using the photon optimizer (PO) version 15.6 and the Acuros XB dose calculation algorithm. Comparisons were made between three methodologies: “Conventional,” “Skin Sparing,” and “SMART.” These represent clinical plans optimized per current protocol, a plan with emphasis placed on optimizing the skin sparing structure (to be defined later), and a plan with emphasis placed on both the skin sparing structure and the oral cavity, respectively. The three techniques were planned and compared to evaluate the effect on both sparing of the organs at risk (OARs) as well as the coverage of the planning target volumes (PTVs). The planner was not consistent for all patients, as the original clinical plan (with variation in planner) was used as a basis for comparison and further optimization. The low dose planning target volume (PTV Low) was prescribed 56 Gy in 35 fractions and included the neck in all cases. The high dose PTV (PTV High) was prescribed 70 Gy in 35 fractions in all cases.

The conventional technique is a copy of the clinical plan, and an objective template was created from this plan for subsequent optimizations. The original objectives were set with the intent to spare normal structures to levels acceptable by current clinical protocols. All target structures were trimmed 3 mm from the body surface. For evaluation purposes a skin structure was created as a 0.5 cm wall structure from the body contour. It was limited to 1 cm superior and inferior to the low dose planning volume, laterally to between the clavicles and posteriorly to the top of the headrest for each patient.

A skin sparing structure was created in a 2‐step process. The total skin was first cropped to include only contours within 4 cm of the low dose planning volume. To avoid underdosing the targets, the skin sparing structure was cropped by 1 cm from the primary target volume (PTV High) and 2 mm from the elective nodal target (PTV Low). The upper optimization objectives used for the skin sparing structure were 50 Gy to 0%, 30 Gy to 5%, and 10 Gy to 50%, all with a priority of 100%. The optimization was run only twice without modification in order to keep the number of iterations consistent between patients. This plan generated the data in the “Skin Sparing” technique.

**FIGURE 1:**
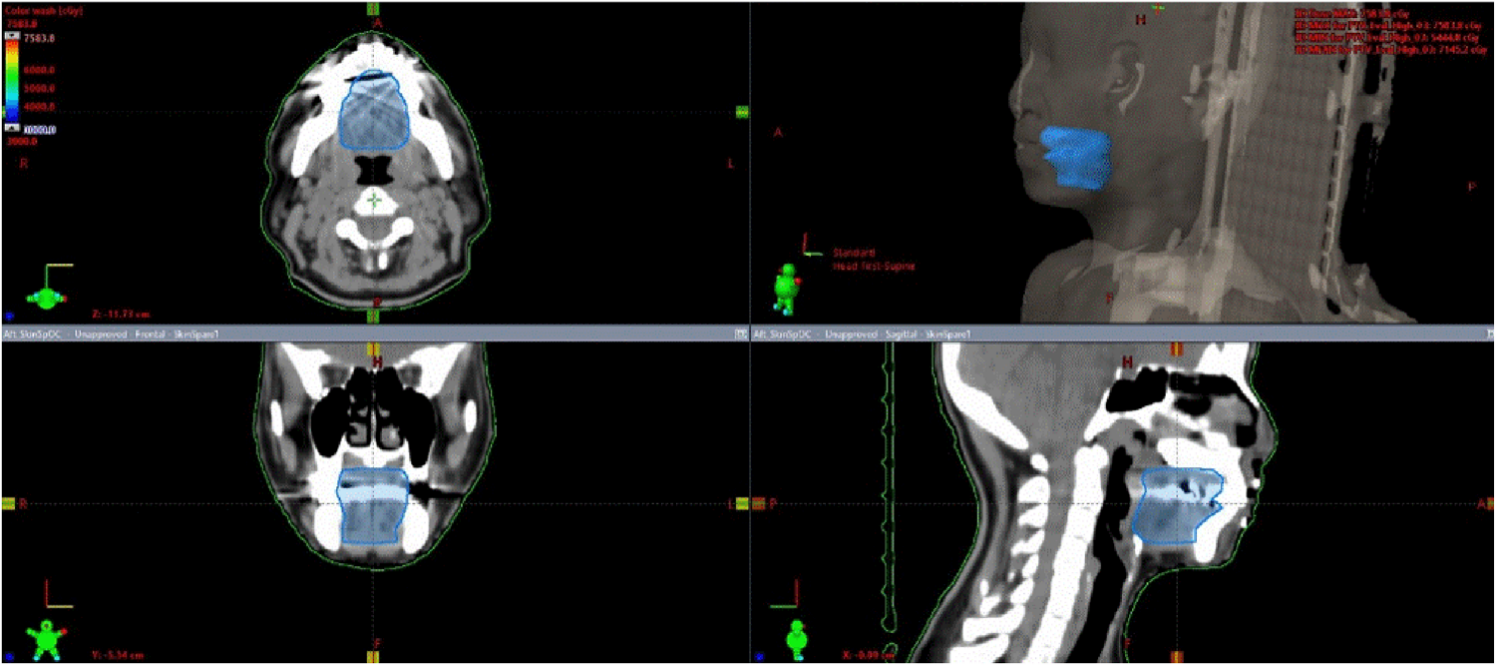
Axial, coronal, and sagittal views of the oral cavity organ at risk contour.

To reduce dose specifically to the oral cavity while still sparing the skin, the oral cavity structure was reprioritized using the following objectives: 50 Gy to 5%, 34.5 Gy to 15%, 20.5 Gy to 35%, and 12.5 Gy to 80% with a priority of 80% and added to the plan described in the “Skin Sparing” technique. These plans were used to generate the data in the “SMART” technique. The oral cavity was defined as a composite structure with the following limits: cranially, the hard palate mucosa and mucosal reflections near the maxilla; caudally, the base of tongue mucosa to the level of the split in the mylohyoid muscles and anterior belly of the digastric muscles; anteriorly, the inner surface of the mandible and maxilla; and posteriorly, the anterior borders of the soft palate, uvula and more inferiorly the base of tongue. Laterally the limit was the inner surface of the mandible and maxilla (Figure 1). Important dose metrics were compared between the three techniques, and statistical significance was determined using analysis of variance, utilizing a Bonferroni correction, to account for multiple pairwise comparisons. To identify which mucosal structure would be the most meaningful to serve as the primary structure for mucosal avoidance, we correlated the maximum toxicity grade experienced during treatment to the 20%, 50%, and 80% of the volume of the oral cavity and midline mucosa.

The primary outcome of the study was to determine if SMART radiotherapy can lower the average dose to the skin that is not adjacent to the high dose planning target volumes, while maintaining acceptable coverage. The secondary outcomes were average reduction in dose to the oral cavity, and mean and maximum doses to brainstem, spinal cord, parotid glands, and larynx. Statistical analysis was conducted using SAS statistical software version 9.4 (SAS Institute, Cary, NC).

## 3 RESULTS

Regarding the CTCAE v5.0 toxicity scoring, there were moderately high correlations between dose to oral cavity and maximum grade experienced during radiotherapy. The Spearman correlation coefficient for dose to 20%, 50%, and 80% of the volume of oral cavity was 0.5 (*p* = 0.048), 0.64 (*p* = 0.007), and 0.62 (*p* = 0.010), respectively. With respect to correlation of maximum grade of radiation dermatitis, there was no correlation between maximum dose, mean dose, nor D80%, for the skin sparing structure. There was, however, correlation between the D20% for the skin sparing structure and maximum grade of radiation dermatitis, with a Spearman correlation coefficient of 0.58 (*p* = 0.0177).

In our cohort of 16 patients, the maximum radiation dermatitis grade during radiotherapy was Grade 1 in 13.3%, Grade 2 in 80%, and Grade 3 in 6.7%. The maximum grade of oral mucositis during radiotherapy was 0 in 13.3%, Grade 1 in 53.3%, Grade 2 in 26.7%, and Grade 3 in 6.7%.

From the treatment planning comparison, the average maximum dose to the skin spare structure was reduced from 64.2 Gy in the conventional plans, to 56.6 and 55.9 Gy (*p* < 0.0001), for both comparisons to the conventional plan) for the Skin Sparing and SMART plans, respectively. The average mean dose to the skin spare structure was reduced from 26.7 Gy in the conventional plans, to 20.0 and 20.2 Gy (*p* < 0.0001), for both comparisons to the conventional plan) for the Skin Sparing and SMART plans, respectively. For the clinically relevant metric, the D20 of the skin sparing structure, there was significant reductions in both the skin sparing plan and SMART plans, reducing the average doses from 39.28 Gy in the conventional plan, to 30.22 Gy in the skin sparing plan, and 30.44 Gy in the SMART plan (*p* value for both pairwise comparisons < 0.0001).

**FIGURE 2:**
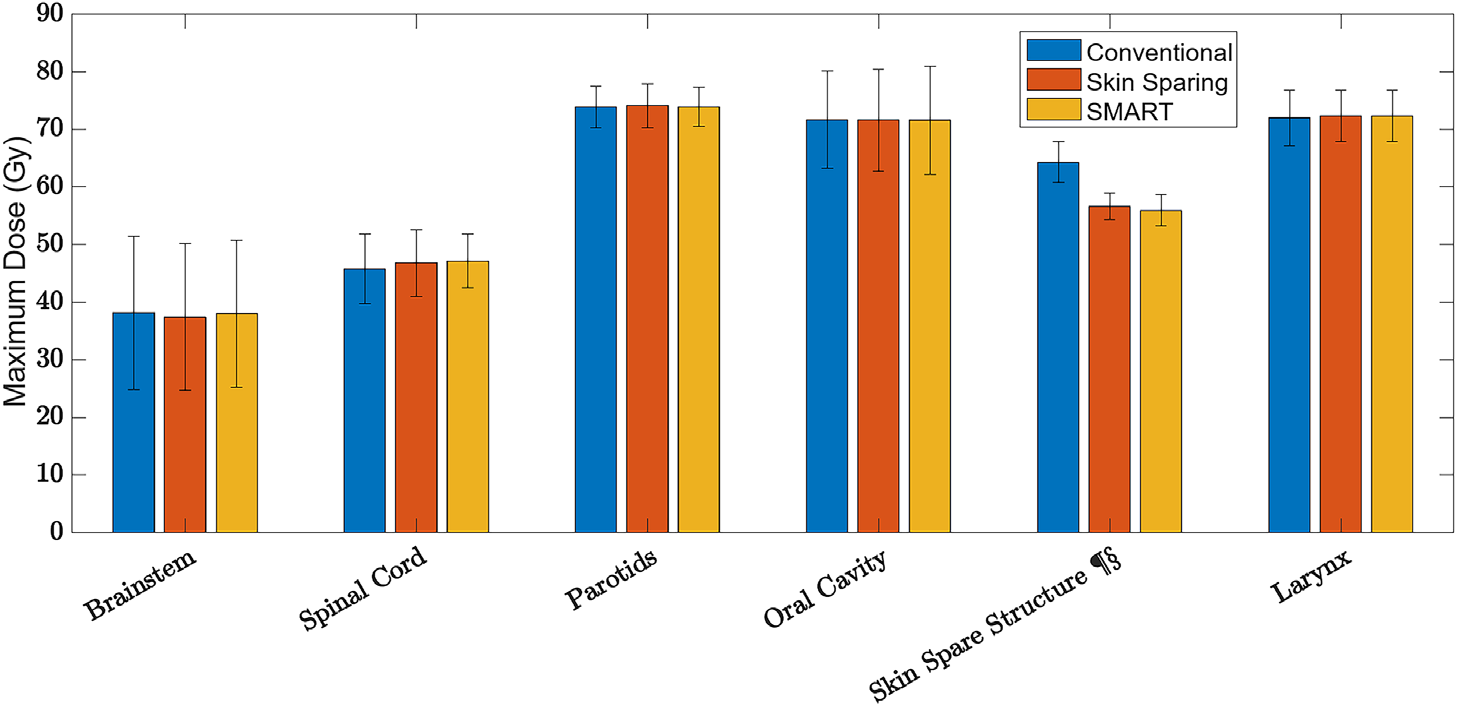
Maximum dose comparison between the conventional (blue), skin sparing (orange), and SMART (yellow) treatment planning techniques for all sixteen patients in the comparison population. The error bars show the standard deviation, and the symbols indicate that the comparison showed statistical significance at *p* < 0.05 where ¶ is the comparison between conventional and Skin Sparing, § is the comparison between conventional and SMART, and † is the comparison between Skin Sparing and SMART.

**FIGURE 3:**
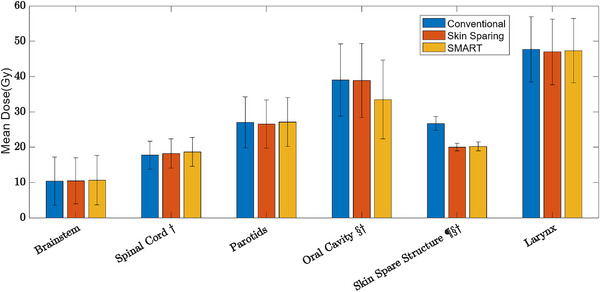
Mean dose comparison between the conventional (blue), skin sparing (orange), and SMART (yellow) treatment planning techniques for all sixteen patients in the comparison population. The error bars show the standard deviation, and the symbols indicate that the comparison showed statistical significance at *p* < 0.05 where ¶ is the comparison between conventional and Skin Sparing, § is the comparison between conventional and SMART, and † is the comparison between Skin Sparing and SMART.

With addition of the oral cavity avoidance in the SMART plans, the mean dose was reduced from 39.0 and 38.9 Gy in the conventional and Skin Sparing techniques, respectively, to 33.5 Gy in the SMART technique. Maximum doses (Figure 2) and mean doses (Figure 3) were compared between the most pertinent OARs. Statistical significance was found (*p* < 0.05) between the maximum dose between conventional and Skin Sparing techniques (denoted by ¶) in only the Skin Spare structure. Between the conventional and SMART techniques (denoted by §) a borderline significance was found for the spinal cord structure (SMART maximum cord dose 47.12 Gy vs. 45.76 Gy for the conventional plan, *p* = 0.069). For mean dose, between conventional and skin sparing techniques, only the skin spare structure was significant. Between conventional and SMART techniques, the oral cavity and skin spare structure were significant. Comparing the conventional technique to both the Skin Sparing technique and the SMART technique (denoted by †) the mean spinal cord dose showed significance (17.74 Gy vs. 18.22 Gy vs. 18.67 Gy, *p* = 0.0001 for SMART vs. conventional, *p* = 0.0315 for skin sparing vs. conventional).

**FIGURE 4:**
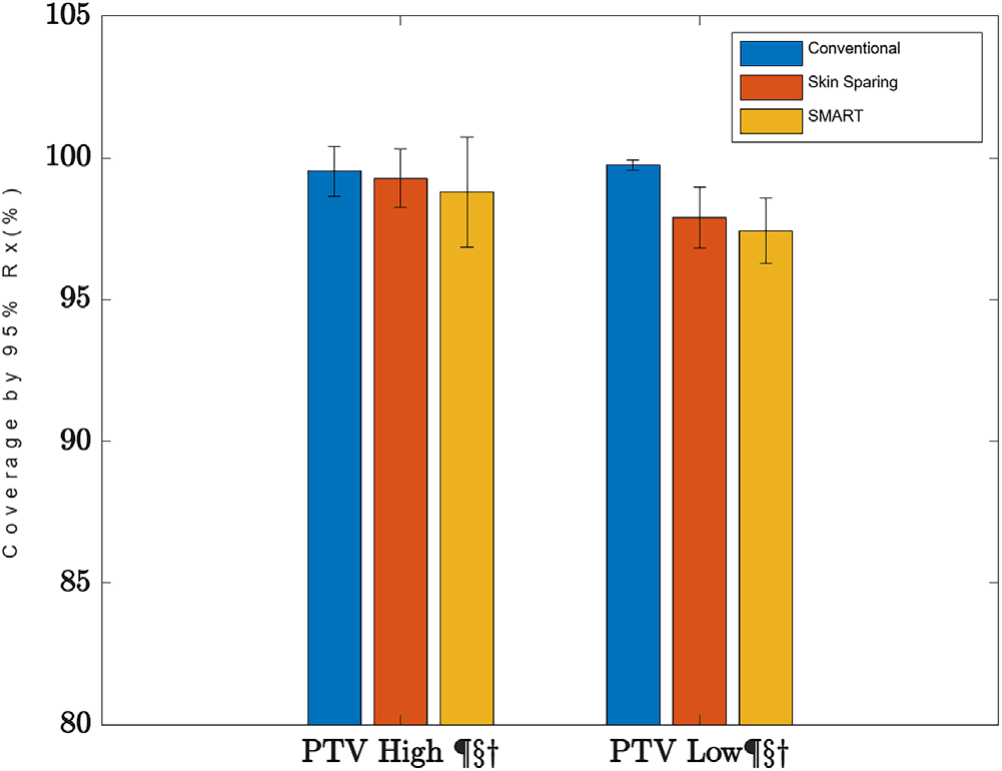
Percent of the PTV High and PTV Low covered by their respective 95% of the prescription dose. The error bars indicate standard deviation, and the symbols indicate that the comparison showed statistical significance at *p* < 0.05 where ¶ is the comparison between conventional and Skin Sparing, § is the comparison between conventional and SMART, and † is the comparison between Skin Sparing and SMART.

There was a slight reduction in PTV_High coverage by the 95% isodose in the SMART plans (99.52% vs. 98.79%, *p* = 0.0073), and a similar slight reduction in PTV_Low coverage by the 95% isodose by both the skin sparing and SMART plans (99.74% vs. 97.89% vs. 97.42%, *p* = < 0.0001) (Figure 4).

**FIGURE 5:**
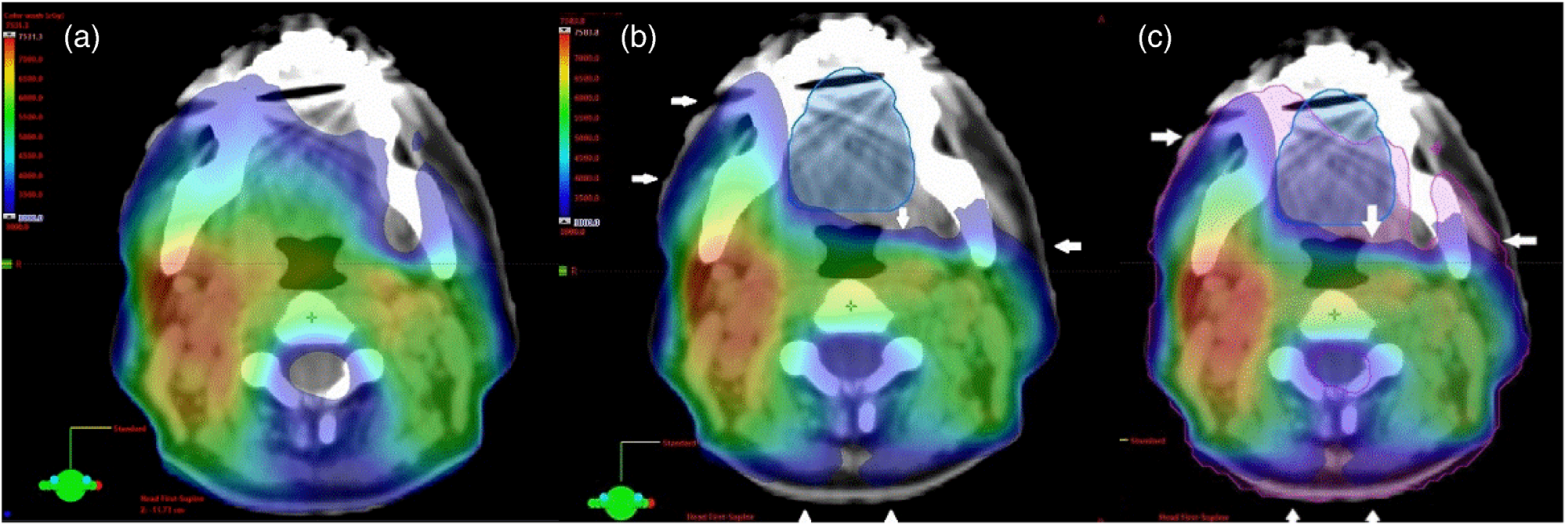
Axial view of the dose distribution from the treatment planning system. The dose wash displayed illustrates the doses above 3000 cGy for the same axial position of the conventional plan (a) and SMART plan (b) with white arrows indicating the areas of increased sparing. (c) An overlay of the two plans on the same CT slice. The pink contour represents the 3000 cGy wash from Plan A.

**FIGURE 6:**
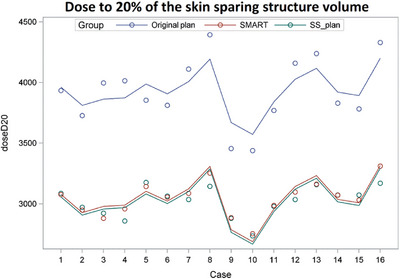
Comparison of D20 for skin sparing plan (SS_plan) and SMART plan (lower green and red line), to D20 for original plan (upper blue line), by case, showing significant reductions in the average D20 (30.22 Gy vs. 30.44 Gy vs. 39.28 Gy, *p* < 0.0001 for both comparisons).

In terms of predicting whether the reduction in dose to oral cavity or skin will translate into clinically meaningful changes, or not, the average oral cavity dose in patients with only Grade 1 mucositis was 35 Gy. The SMART plans were able to achieve an average dose of 33.5 Gy to the oral cavity, so we believe that a clinically meaningful optimization of oral cavity dose can be achieved, without significant reduction in PTV coverage (Figure 5). Similarly, using the D20% metric for the skin sparing structure, these plans yielded an average D20% of 30.4 Gy, compared to the average D20% of 36.1 Gy in patients experiencing a maximum of Grade 1 radiation dermatitis (Figure 6).

## 4 DISCUSSION

Using our methodology, it appears the SMART H&N radiotherapy planning technique can reduce the average dose to both the skin in the lower dose PTV, and to the oral cavity, with acceptable PTV coverage and dose to critical OARs, like parotids, brainstem, and spinal cord. The trade‐off to reduce acute skin and mucosal toxicity was numerically higher mean spinal cord dose (18.67 Gy vs. 17.74 Gy, *p* < 0.0001) and a slight reduction in PTV coverage, which we feel are not clinically significant. The reductions in dose to skin and oral cavity do predict that the organ sparing that could be achievable is like the average doses that are seen with CTCAE v5.0 grade 1 toxicity. Given our results, the numerical reduction in average doses to skin and oral cavity (Figure 5) should be sufficient to result in a clinically meaningful reduction in the skin and mucosal toxicity that patients experience during treatment.

Despite the increased complexity of SMART radiotherapy, mainly with regards to the construction of the skin‐sparing avoidance structure, we do not see a clinically relevant reduction in PTV coverage. Limitations of our study include a small sample size, and that the toxicity assessments were rated by care providers, as opposed to patient‐reported outcomes, which can introduce bias. However, the toxicity assessments were utilized mainly to determine which mucosal OAR to select for the purposes of the study, and not how to optimize the SMART radiotherapy planning.

Previous studies have shown similar results, except they focused exclusively on skin sparing. A dosimetric study of 10 patients, with particular attention to skin sparing yielded an average reduction of V35 Gy of 10.3% in the skin‐sparing plans versus the conventional plans.^5^ A previous study by Lee,^4^ showed that in four patients when attention was paid to skin‐sparing, the volume of skin that received 45 Gy could be reduced by 20%. Our results are in keeping with these studies. As for sparing of the oral cavity, we were able to achieve an improvement of the oral cavity mean dose to 33.5 Gy, from 39 Gy, which is close to the NRG‐HN002 constraint. It is important to point out that the dose utilized in our study was 70 Gy in 35 fractions, whereas NRG‐HN002 utilized a dose of 60 Gy in 30 fractions, as part of a de‐intensification study in patients with p16+ve tumors. They reported a 21.1% rate of grade 3 or greater mucositis, compared to our cohort which reported a 6.7% grade 3 or greater mucositis, suggesting our center is already achieving reasonable degrees of oral cavity sparing. Both these studies compare favorably to the early days of SS‐IMRT that reported 25%–80% grade 3 or greater mucositis^7^, ^10^, ^11^

Though the reduction in doses can be theoretically achieved, what is not known is what target dose and volume will result in clinically significant improvements in toxicity and patient reported outcomes.

## 5 CONCLUSION

SMART radiotherapy appears feasible and practical in this dosimetric study. To demonstrate definitively that our methodology is robust, and clinically meaningful, we feel a clinical trial of conventionally planned versus SMART planned radiotherapy is warranted for patients receiving radiotherapy for the curative treatment of head and neck cancer, in the context of a randomized trial. Correlation of dose and volume to patient reported toxicity will shed light on what normal tissue organ constraints will achieve less toxicity, while maintaining high rates of cancer control.

## AUTHOR CONTRIBUTIONS

Cheryl Anderson and Derek Wilke designed the study. Cheryl Anderson and Lee MacDonald acquired the data for the study. Data analysis was performed by Lee MacDonald and Derek Wilke. All of the authors reviewed the manuscript and gave their feedback on the findings.

## CONFLICT OF INTEREST STATEMENT

The authors declare no conflicts of interest.
